# WorldFlora: An R package for exact and fuzzy matching of plant names against the World Flora Online taxonomic backbone data

**DOI:** 10.1002/aps3.11388

**Published:** 2020-09-25

**Authors:** Roeland Kindt

**Affiliations:** ^1^ Tree Productivity and Diversity World Agroforestry P.O. Box 30677‐00100 Nairobi Kenya

**Keywords:** biodiversity informatics, online flora, plant name identification, species name matching, spelling errors, taxonomic databases

## Abstract

**Premise:**

The standardization of plant names is a critical step in various fields of biology, including biodiversity, biogeography, and vegetation research. The WorldFlora package is introduced here to help achieve this goal by matching lists of plant names with a static copy from World Flora Online (WFO), an ongoing global effort to complete an online flora of all known vascular plants and bryophytes by 2020.

**Methods and Results:**

Based on direct and fuzzy matching, WorldFlora inserts matching cases from the WFO to a submitted data set containing taxonomic names. The results and success rates for selecting the expected best single matches are presented for four data sets, including two data sets used in recent comparisons of software tools for correcting taxon names.

**Conclusions:**

WorldFlora offers a straightforward pipeline for semi‐automatic plant name checking. For the four data sets, the success rate of credible matches ranged from 94.7% to 99.9%.

The scientific names of organisms and the higher groups into which they are classified are key identifiers of the world’s biodiversity (Rees, 2014). Assigning a species identity to an organism is essential in a wide array of disciplines including ecology, conservation, and forestry (Tyrell, 2019); therefore, synonyms need to be removed from plant species lists to enable the prediction of the total number of vascular, seed, and flowering plant species (Lughadha et al., 2016). Taxonomic uncertainty is one of the major gaps in the plant occurrence data needed for global plant ecological, biogeographical, and conservation applications (Meyer et al., 2016), and misspellings of scientific names can lead to failures to retrieve data from global databases that encompass millions of species (Boyle et al., 2013). Matching alternative species names and resolving synonyms was, for example, essential for the development of the Agroforestry Species Switchboard, a portal that currently documents the presence of 172,395 plant species across 35 web‐based information sources through 307,404 hyperlinks (Kindt et al., 2019). If not corrected, the lack of standardization of names can lead to inflated estimates of species richness; for example, a revision of a checklist of 11,675 Amazonian tree species through a taxonomic vetting process resulted in an updated list of 10,071 species, representing a “loss” of around 15% of taxa (ter Steege et al., 2019). Issues of misspellings and unresolved synonyms increase the risks of erroneous scientific conclusions, such as the misidentification of medicinal applications in plants, with potentially serious consequences (Sharma et al., 2019).

Target 1 of the Global Strategy for Plant Conservation is to complete an online flora of all known plants by 2020. To achieve this goal, the World Flora Council was formed, including 36 participating institutions (Miller and Ulate, 2017). Building on The Plant List (http://www.theplantlist.org), which became static in 2013, the World Flora Online’s (WFO) taxonomic backbone is actively curated by taxonomic specialists and Taxonomic Expert Networks (Palese et al., 2019). Static copies of the taxonomic backbone data are available from the WFO website (http://www.worldfloraonline.org/downloadData [accessed 11 August 2020]). The WorldFlora package is introduced here as a tool to match lists of taxa with the WFO taxonomic backbone; at the time of publication, it was the only package known to do this. An overview of the fields of the WFO backbone data can be obtained in WorldFlora using the following script: library(WorldFlora); data(WFO.example); and WFO.example. This overview is also available in Appendices S1–S5, because WorldFlora includes all fields from the WFO backbone data in the output of its main functions.

## METHODS AND RESULTS

WorldFlora is an R package (R Core Team, 2019) that matches a list of plant names with a static version of the WFO taxonomic backbone data (examples presented herein used version 2019.05 from 17 May 2019). Data sets can be imported in R in a wide variety of formats, with various input options available through the graphical user interface of R‐Commander (Fox, 2005). Exact matching can be examined for the entire plant name or simultaneously for the genus, species, and infraspecific levels with the WorldFlora::WFO.match function. Fuzzy matching is implemented for the full plant name using WFO.match, calculating the Levenshtein Distance (LD) via the R functions base::agrep (a user can modify its argument of max.distance from the default 0.1) and utils::adist. The LD measures single‐character substitutions, insertions, or deletions, each contributing a value of 1 to the total distance. The Taxamatch algorithm (Rees, 2014), also implemented in the Taxonomic Name Resolution Service (TNRS) (Boyle et al., 2013), uses the modified Damerau–Levenshtein distance where transpositions are given lower weights (2 in the example of “vecusilosus” and “vesiculosus”) than the weights of substituting each character individually (4 in the same example). Users can limit the number of matches to those with the smallest LD by setting the argument Fuzzy.min = TRUE as the default setting. The WorldFlora::WFO.one function finds single matches for each submitted plant name by filtering records by accepted name and synonym status. Information about the rationale for selection is given in a separate column in the output. Where multiple candidates remain, the function selects the match with the smallest WFOID field in the taxonomic backbone. Successful matches performed by WorldFlora are limited to the WFO’s scope of vascular plants and bryophytes, similar to software packages that use The Plant List such as Taxonstand (Cayuela et al., 2012). Therefore, users should ideally not attempt to resolve names from organism groups that are not covered, such as algae, fungi, or lichens (Wagner, 2016).

Four data sets were used to check the performance of WorldFlora and to describe some of its features. The first is a random subset of 1000 species selected from the GlobalTreeSearch database (Beech et al., 2017) (version 1.3 accessed from https://tools.bgci.org/global_tree_search.php [accessed 11 August 2020]). Of these, 957 species were matched directly (Table 1); the remaining 43 were matched by the fuzzy algorithm. Where several matches were retrieved, the first option of finding the single best match was achieved by selecting the record with the smallest LD between the submitted and matched authority (selecting the best author match is the default option for WorldFlora::WFO.one if the Auth.dist variable is declared). This option resulted in 24 single matches, including 21 matches with an LD of zero between authorities (e.g., the selection of *Bauhinia grandifolia* (Bong.) D. Dietr. and rejection of *Bauhinia grandifolia* Steud.; see Appendix S1). The remaining five single matches were based on not selecting a synonym name, with four of those matches having an LD of zero between authorities (e.g., by selecting *Xylosma intermedia* (Seem.) Griseb. and rejecting the synonym *Xylosma intermedia* (Seem.) Triana & Planch.). For 957 species, the name retrieved by WorldFlora was exactly the same as the submitted name (classified as “correct” in Table 1). Among the names that were not matched exactly, three were spelling variants (*Callianthe sylvatica* vs. *Calanthe sylvatica*, *Euodia cuspidata* vs. *Evodia cuspidata*, and *Guatteria moralesii* vs. *Guatteria moralesi*). Five names would have been matched if a scientist familiar with the revision of the *Acacia* genus (Brummitt, 2004) had substituted the genera *Senegalia* or *Vachellia* with *Acacia*, resulting in a tally of 965 (957 + 3 + 5; Table 1) credible matches that can be achieved using WorldFlora in a typical supervised workflow of 1000 species. Of the 35 species without credible matches, 28 were partially matched at the genus level through the default option of argument Fuzzy.one that searched for matches only for the first word, in case the full name yielded no matches (Table 2 provides an overview of various function arguments).

**Table 1:** Results of matching plant names from four data sets. The difference between correct and credible matches is clarified in the text.^a^

Plant taxa counts	GTS	CTTS	Wagner	SALVIAS
Taxa	1000	1741	600	1000
Unique direct matches	930	1513	0	694
Multiple direct matches	27	125	0	40
Unique fuzzy matches	41	100	491	232
Multiple fuzzy matches	2	3	41	22
No match	0	0	68	12
Correct matches	957	1728	500	951
Credible matches	965	1740	568	975

Note

CTTS = commercial timber tree species data set; GTS = Global Tree Search data set; SALVIAS = testing data set from Boyle et al. (2013); Wagner = combined testing data set from Wagner (2016).

^a^ Details about access, data manipulation, outputs, and R scripts are available in Appendices S1 (GTS data set), S2 (CTTS data set), S3 (Wagner data set), and S4 (SALVIAS data set).

**Table 2:** Overview of some of the WorldFlora::WFO.match arguments.

Argument	Details
Authorship	If this variable is found in the submitted data, the result will include a column of “Auth.dist” with the LD between the submitted and matched naming author.
acceptedNameUsageID.match	In the default setting, where the WFO includes an acceptedNameUsageID (typically indicating the accepted name for a synonym), then the matched details in the results will show the accepted name, and the columns “Old.status”, “Old.ID”, and “Old.name” will show those of the first match.
Fuzzy.max	The maximum number of names matched by the fuzzy algorithm
Fuzzy.min	In the default setting (TRUE), only the matches with the smallest LD are retained.
Fuzzy.two	Flags (TRUE/FALSE) whether the match was obtained for only the first two words of the submitted name.
Fuzzy.one	Flags (TRUE/FALSE) whether the match was obtained for only the first word of the submitted name.
spec.name.nonumber	“Number.detected” flags (TRUE/FALSE) whether the submitted name contained a number. If that was the case, a match was searched only for the first word of the submitted name.
spec.name.tolower	Converts all characters of the submitted name to lowercase, except the first character
spec.name.nobrackets	“Brackets.detected” flags (TRUE/FALSE) whether the submitted name contained a bracket. If that was the case, the function searched only for the part of the submitted name before the bracket.

The second data set used as a case study was a working list of 1741 commercial timber tree species (Mark et al., 2014). The names were matched directly for 1638 taxa and by fuzzy matching for the remaining 103 names (Table 1). Of the fuzzy matches, 88 had an LD of 1 due to a space character at the end of the submitted name. The WFO.one function used the criterion of the smallest WFOID for 14 names (e.g., selecting wfo‐0000913932: *Scottellia coriacea* A. Chev. ex Hutch. & Dalziel and rejecting wfo‐0000913938: *Scottellia coriacea* A. Chev.), but in only one case were the candidate species different (selecting synonym wfo‐0000416717: *Ulmus glabra* and rejecting synonym wfo‐0000475766: *Planera aquatica* for the submitted *Ulmus campestris*). For 28 species where the original data set had included a synonym name between brackets, it was confirmed that this was indeed the accepted synonym name (e.g., *Dysoxylum euphlebium* is a synonym of *Dysoxylum alliaceum*). Among the 13 species where the submitted and retrieved names were not identical (not “correct” as in Table 1), 12 were spelling variants such as *Pinus englemannii* vs. *Pinus engelmannii* or *Sequoiadendron gigantum* vs. *Sequoiadendron giganteum*. *Populus canescens* was matched by its hybrid name *Populus ×canescens*, correctly reflecting that the species is now accepted to be a hybrid of *Populus alba × Populus tremula* (Tutin et al., 1993). The only species without a credible match was *Upuna borneensis* (Randi et al., 2019), which was matched by *Upuna boreensis*.

The third data set that was tested was a combination of three data subsets initially created to compare different software packages for correcting plant names (“Wagner”; Wagner, 2016). The argument Fuzzy.max was increased to 2500 because an initial run of WFO.match could not resolve many of the names submitted at the genus rank. As the testing procedure involved deleting the last character of each species name in a list of species names, all the matches were fuzzy, as might be expected (Table 1). Of the 600 species in this data set, 100 names were not identical to the expected names (not “correct” in Table 1). Of these, 60 were the names of algae, fungi, and lichen species outside the scope of WFO and its predecessor, The Plant List. Five of the incorrect names were spelling variants, one was an interspecies hybrid (*Carex acuta* × *elata*), and two were matched as varieties rather than the submitted subspecies names (*Keckiella antirrhinoides* var*. microphylla* for *Keckiella antirrhinoides* subsp. *microphylla* and *Saxifraga adscendens* var*. oregonensis* for *Saxifraga adscendens* subsp. *oregonensis*). No acceptable matches were found for seven of the remaining 32 species because the submitted family was not included in the WFO (i.e., Aceraceae, Najadaceae, Punicaceae, Taccaceae, Theophrastaceae, Tiliaceae, and Vittariaceae); none of these families were retained in the fourth update of the classification of the angiosperm orders and families published by the Angiosperm Phylogeny Group (Chase et al., 2016). Seven were names for which the number of fuzzy matches was too high to retrieve the genus name, while there were nine names where a submitted hybrid was matched by a non‐hybrid name. In three cases, mismatches resulted from selecting the record with the smallest WFOID from otherwise valid fuzzy alternatives (selecting *Lomatia* R. Br. [Proteaceae] not *Lomatium* Raf. [Apiaceae], *Placea* instead of Poaceae, and *Sabicea* instead of Sabiaceae). With an overall matching success of 87.6% (219 out of 250 species) for spellchecking tests across different taxonomic ranks, WorldFlora performed better than the Global Names Resolver (GNR; 74% of species matched), which was the best software in the comparisons undertaken by Wagner (2016). With 99.6% correct matches in comparisons across different geographic regions (only *Leptinella conjuncta* was not matched of the 250 submitted names), WorldFlora also marginally outperformed GNR as the best software, with GNR giving 99.2% correct matches in the earlier comparison (Wagner, 2016).

The fourth data set that was analyzed, “SALVIAS,” is a list of 1000 plant names used in a previous comparison of the TNRS with other online tools for the automated standardization of plant names (Boyle et al., 2013). The same data set was also used in a more recent evaluation study (Sharma et al., 2019). This data set offers various challenges to plant name checking, such as the inclusion of names in capital letters. This particular challenge is handled by WFO.match by option spec.name.tolower=TRUE, whereby submitted names are converted to lowercase, except for the first character. The challenge of handling semi‐standardized qualifiers that are used in plant names (e.g., “cf.” to indicate that not all of the diagnostic characters correspond to a given species, or “aff.” to indicate that a specimen has some affinity but is not identical to a known species) is handled by deleting these characters from the name. Taxamatch (Rees, 2014) and the TNRS (Boyle et al., 2013) use a similar approach. The default list of qualifiers used by WFO.match was derived from descriptions provided by Sigovini et al. (2016), also including qualifiers such as “sp.”, “indet.”, and “nom. nov.”. As WFO.match expects that the submitted plant name does not include the naming authority, if no matches are found with the submitted name that include the authority, a search is performed for the first two words of the submitted name, which is expected to correspond to the genus and species names (default option for argument Fuzzy.two). If the search still does not find a name match, then a match is attempted for the first word only (default option for argument Fuzzy.one). Names that contain brackets, possibly at the beginning of the authority name, are stripped from the entire part starting with the bracket (default option of spec.name.nobrackets). Names that contain numbers are searched only for the first word, with the remaining part of the submitted name suspected to correspond to an unidentified species (default option of spec.name.nonumber).

An initial run of WorldFlora.match with SALVIAS yielded 25 names with no match. A visual inspection of these unmatched names revealed cases where the qualifier “cf” was used instead of the standard “cf.”, or where “aff” was used instead of “aff.”. Simulating how an actual semi‐automatic pipeline of plant name checking would work, incomplete qualifiers were manually replaced by the correct qualifier that is recognized in the argument sub.pattern. Likewise, the argument Fuzzy.max was increased to 2000, as the output of WorldFlora.match indicated a series of names where the number of fuzzy matches was above the default 250, with a maximum of 1974 fuzzy matches for the submitted “Miconia”. The second run of WorldFlora.match with the modified names and arguments resulted in 734 directly matched names and 254 names with fuzzy matches (Table 1). Directly comparing the final subset of names obtained via WorldFlora.one with those obtained using the TNRS (http://tnrs.iplantcollaborative.org/TNRSapp.html [accessed 12 December 2019]) with default settings showed 951 identical names (“correct” in Table 1). Among the 49 names that were not identical, eight were spelling variants such as *Commiphora laxecymigera* vs. *Commiphora laxicymigera*. For 16 of these names, WorldFlora resulted in a more credible match than the TNRS; for example, the submitted Asteraceae and Fabaceae families were correctly matched by WorldFlora, but matched incorrectly as the Asteliaceae or Fagaceae by the TNRS. The TNRS provided a partial match of the submitted *Solanum schlechtendali* to *Solanum*, whereas WorldFlora correctly matched *Solanum schlechtendalianum*. Accepting these credible matches by WorldFlora resulted in the correct matching of 975 names, a number close to the 980 correct matches reported for the TNRS (Boyle et al., 2013). The number of matches was greater than the 950 achieved by Solr‐Plant using the same data set, which performed second best among the comparison of TNRS, Solr‐Plant, Plantminer, and GNResolver (Sharma et al., 2019).

## CONCLUSIONS

The analysis of the four data sets showed that the new package, WorldFlora, correctly matched the majority of submitted plant names with the WFO taxonomic backbone, with success rates comparable to and sometimes better than alternative software packages such as the TNRS or Solr‐Plant. Users are advised not to blindly accept results, however, as limitations in plant name checking software and spelling mistakes in reference databases can result in incorrect matches (Wagner, 2016). Users are therefore especially advised to screen the output of the WorldFlora::WFO.match and WorldFlora::WFO.one functions for information that could indicate possible mismatches, such as a large LD between the submitted and matched plant names, or the justification of a single match based on the smallest WFOID field of the taxonomic backbone. Where possible, users are further advised to compare results obtained between different applications, for example, by submitting names both to WorldFlora and the TNRS, first checking where both software packages agree on the accepted plant name.

As the WFO provides a further development of The Plant List, which became static in 2013, the matching of plant names with WorldFlora is expected to provide results that are more up to date than software packages that rely on The Plant List, such as Taxonstand (Cayuela et al., 2012). To benefit maximally from the current state of knowledge on plant names, users should ensure that they use the most recent version of the WFO backbone database. Because naming authorities are not part of the plant name that is checked, users ideally should submit the plant name and the authority in separate fields to reduce mismatches.

## AUTHOR CONTRIBUTIONS

R.K. conceptualized and wrote this article, including compilation of data sets and analysis of results. He is also the sole author of the WorldFlora and RcmdrPlugin.WorldFlora R packages.

## Supporting information


**APPENDIX S1.** Details of the data, scripts, and WorldFlora results for the analysis of a subset of the GlobalTreeSearch database.

**APPENDIX S2.** Details of the data, scripts, and WorldFlora results for the analysis of a working list of commercial timber tree species.

**APPENDIX S3.** Details of the data, scripts, and WorldFlora results for the analysis of the “Wagner” data set.

**APPENDIX S4.** Details of the data, scripts, and WorldFlora results for the analysis of the “SALVIAS” data set.

**APPENDIX S5.** Scripts and messages obtained during the analysis of the four case studies.

## Data Availability

The WorldFlora package is published under the GNU General Public License (version 2; https://www.gnu.org/licenses/old‐licenses/gpl‐2.0.en.html [accessed 12 August 2020]). The software and related documentation are available for free download from the Comprehensive R Archive Network (https://cran.r‐project.org/package=WorldFlora).
